# Targeting cellular respiration as a therapeutic strategy in glioblastoma

**DOI:** 10.18632/oncotarget.28424

**Published:** 2023-05-04

**Authors:** Enyuan Shang, Trang Thi Thu Nguyen, Mike-Andrew Westhoff, Georg Karpel-Massler, Markus D. Siegelin

**Affiliations:** ^1^Department of Pathology and Cell Biology, Columbia University Medical Center, New York, NY 10032, USA; ^2^Department of Biological Sciences, Bronx Community College, City University of New York, NY 10453, USA; ^3^Department of Neurosurgery, Ulm University Medical Center, Ulm 89081, Germany; ^4^Department of Pediatrics and Adolescent Medicine, Ulm University Medical Center, Ulm 89081, Germany

**Keywords:** glioblastoma, metabolism, lactate, carbon tracing, central carbon metabolism

## Abstract

While glycolysis is abundant in malignancies, mitochondrial metabolism is significant as well. Mitochondria harbor the enzymes relevant for cellular respiration, which is a critical pathway for both regeneration of reduction equivalents and energy production in the form of ATP. The oxidation of NADH_2_ and FADH_2_ are fundamental since NAD and FAD are the key components of the TCA-cycle that is critical to entertain biosynthesis in cancer cells. The TCA-cycle itself is predominantly fueled through carbons from glucose, glutamine, fatty acids and lactate. Targeting mitochondrial energy metabolism appears feasible through several drug compounds that activate the CLPP protein or interfere with NADH-dehydrogenase, pyruvate-dehydrogenase, enzymes of the TCA-cycle and mitochondrial matrix chaperones. While these compounds have demonstrated anti-cancer effects *in vivo*, recent research suggests which patients most likely benefit from such treatments. Here, we provide a brief overview of the status quo of targeting mitochondrial energy metabolism in glioblastoma and highlight a novel combination therapy.

## INTRODUCTION

**Figure 1:**
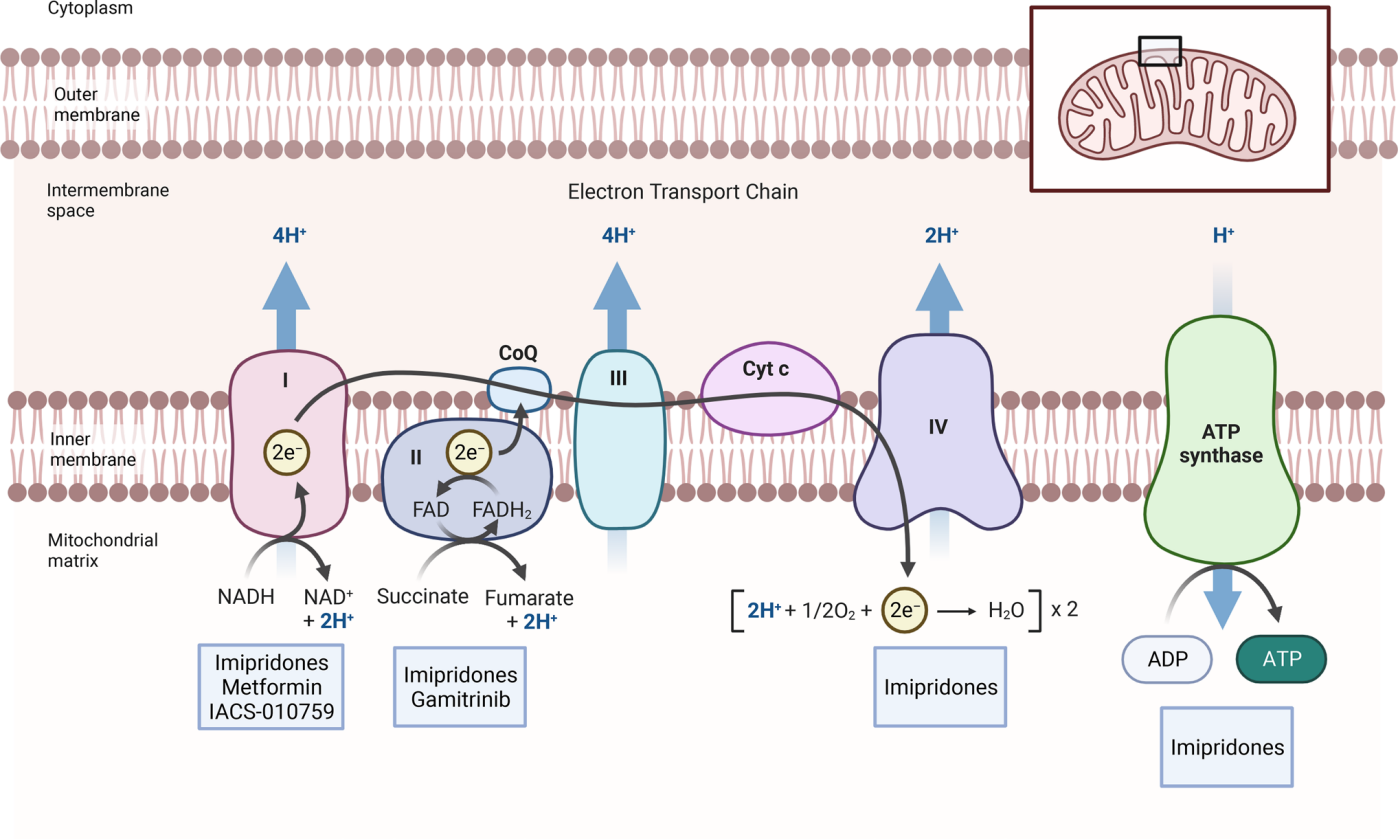
Inhibitors of mitochondrial respiration. The figure shows the respiratory chain with all its complexes. Electrons are received at the level of complex I (NADH_2_) and complex II (FADH_2_), respectively. They are then transferred to complex IV, which mediates the reduction of molecular oxygen to water. An electrochemical gradient is produced by complexes I, III and IV that in turn is utilized by complex V to produce ATP. Several inhibitors are highlighted that target complex I (metformin and IACS-010759), predominantly complex II (gamitrinib) and several complexes (imipridones, CLPP activators).

Mitochondria are cellular organelles that drive biosynthesis and ATP production in tumor cells since they contain the enzymes necessary for cellular respiration and the associated process of oxidative phosphorylation, which is the production of ATP from ADP and inorganic phosphate [1, 2]. Moreover, the matrix of mitochondria houses the TCA-cycle, which operates in close connection with the respiratory chain since it produces reduction equivalents in the form of NADH_2_ and FADH_2_. Both NADH_2_ and FADH_2_ require quick regeneration or oxidation so that the TCA-cycle may maintain its activity. The TCA-cycle receives carbons from a number of different substrates, which includes glucose, fatty acid and amino acids [3]. Glucose is metabolized in the process of glycolysis in the cytosol and yields either pyruvate or lactate. While lactate is released into the microenvironment through the MCT4 or MCT1 transporter, pyruvate is shuttled to the mitochondria to be either used as an anaplerotic substrate for the TCA-cycle or alternatively be oxidized to the key node molecule, acetyl-CoA [4–6]. In addition to these fundamental pathways nucleotide and amino acid synthesis is tied to the mitochondria as well, e.g., pyrimidine biosynthesis. In the setting of amino acids glutamine carbons are introduced into the TCA-cycle as well, which involves the glutaminase reaction [7]. Another potential fundamental fuel to entertain respiration are fatty acids, such as palmitic acid, which are very rich in energy since their degradation involves several rounds of oxidation (within beta-oxidation in the mitochondria) that creates substantial amounts of NADH_2_ and FADH_2_, which in turn can provide their electrons to either complex I or complex II of the respiratory chain and generate ATP (Figure 1). While some cancer cells tend to prefer a glycolytic dominant metabolization pathway with lactate production, others tend to oxidize glucose. Consistently, recent research identified that glioblastomas (GBMs) can be classified by their state of metabolism and in this regard a mitochondrial subtype was described, which apparently displays marked susceptibility against inhibitors of oxidative phosphorylation [8]. Moreover, the difference in fuel utilization is likely to have an impact on response and resistance to therapy. Treatment mediated reprogramming of tumor metabolism is another emerging critical mechanism of resistance to therapy and targeting such aberrations might prove effective as novel therapeutic approaches [9, 10].

### Inhibitors of the TCA-cycle and fatty acid oxidation

It is notable that all major mitochondrial fuel sources may be inhibited by pharmacological inhibitors. For instance, the oxidation of pyruvate (derived from glycolysis) may be blocked by PDHA inhibitors, e.g., CPI-613, which has reached phase III clinical testing [11–13]. In the setting of fatty acid oxidation, there is etomoxir or perhexiline that have been tested in patients before [14, 15]. Recent research from our group provided evidence that CPI-613 interferes with GBM growth *in vitro* and *in vivo* [1]. Similarly, several combination therapies, involving etomoxir, have shown preclinical efficacy [14, 16].

### Inhibitors of the NADH-dehydrogenase (complex I)

Cellular respiration and oxidative phosphorylation are critical drivers of ATP production even in malignant cells. Moreover, cellular respiration is a key event to regenerate NAD and FAD that in turn are necessary to sustain the activity of the TCA-cycle thereby driving fundamental biosynthetic processes, e.g., the production of cytosolic acetyl-CoA, the synthesis of aspartate and others. There are several complexes of the respiratory chain. In this context, NADH-dehydrogenase, complex I, is targeted by metformin, an anti-diabetic drug, as well as by a novel compound, called IACS-010759 [17], which appeared to suppress the levels of the amino acid aspartate, which is pivotal for pyrimidine synthesis. Moreover, aspartate supplementation appears to rescue from inhibition of complex I. Similarly, transfection of the Ndi1 yeast cDNA reverses the reduction of viability of cancer cells induced by metformin and IACS-010759 [17], which demonstrated efficacy in preclinical models of leukemia and GBM [17]. A recent intriguing finding relates to the ability of metformin to suppress PD-L1 levels by its ability to interfere with energy metabolism, linking the respiratory chain with the immune system and microenvironment [18, 19]. While these findings were seen in a limited number of model systems it remains to be determined how broad the impact of metformin on PD-L1 is in other tumor models, including GBM.

### BH3-mimetics as indirect inhibitors of cellular respiration

While the fundamental pathways of metabolism are critical for cancer cell survival, tumor mitochondria orchestrate another important aspect, which is intrinsic apoptosis [20]. Ironically, cytochrome-c, which is part of the respiratory chain, has a main role in facilitating intrinsic apoptosis by activation of the apoptosome in the cytosol. The release of cytochrome-c from the mitochondria to the cytosol is strictly controlled by the Bcl-2 family of proteins, BAX and BAK. In turn, these proteins are sequestered by anti-apoptotic Bcl-2 family proteins, Bcl-2, Bcl-xL and Mcl-1.

In this regard, a drug class, called BH3-mimetics, was developed to facilitate the release of BAX and BAK from either Bcl-2 and Bcl-xL [21, 22]. More recently, Mcl-1 targeting BH3-mimetics has been described as well, establishing the unique and potent opportunity to combine two different classes of BH3-mimetic that in turn exert a durable release effect on BAK [23]. The first engineered BH3-mimetic was ABT-737 published in 2005, which had suboptimal pharmacokinetics (e.g., no feasibility of oral administration). While at the first glance these compounds appear to exert no direct effect on metabolism it seems likely that they regulate it through their effects on anti-apoptotic Bcl-2 family members, which modulate cellular respiration [24].

### Gamitrinib is a TRAP1 inhibitor that blocks the function of complex II of the respiratory chain

Aside from BH3-mimetics, mitochondrial Hsp90 antagonists induce cell death as well [25, 26]. These compounds target both TRAP1 and mitochondrial Hsp90 and thereby preferentially disrupt mitochondrial respiration in cancer cells. The name of these reagents is gamitrinib (geldanamycin (GA)-mitochondrial matrix inhibitors). Conceptionally, the first mitochondrial Hsp90 inhibitor was a peptidomimetic, called shepherdin, which was discovered based on the interaction between Hsp90 and the inhibitor of apoptosis protein, survivin [27]. Shepherdin caused substantial cell death in a variety of cancer cells, including both leukemia and solid tumor lines [28]. While certain off-target effects might not be entirely excluded, it appears that succinate dehydrogenase is one of the key targets affected by gamitrinib [29, 30]. Consistently, several studies have shown that gamitrinib potently suppresses the oxygen consumption rate of various tumor cells, including GBM [16, 31]. Due to its profound effect on metabolism, gamitrinib elicited a mitochondrial unfolded stress response with up-regulation of CEBP/beta and CHOP that in turn suppressed NF-κB activity in GBM cells. In turn, loss of NF-kb function sensitized GBM cells to death-receptor mediated apoptosis (extrinsic) [25]. Several preclinical studies have shown efficacy in GBM model systems [16, 31]. It should be noted that gamitrinib synergized with BH3-mimetic to kill GBM cells *in vitro* and in an orthotopic patient-derived xenograft model of GBM in mice [23]. While this study focused predominantly on glioma model systems it is important to highlight the fact that other tumor entities were affected as well, involving other solid malignancies and leukemias. Currently, gamitrinib is being assessed in a clinical phase I trial in patients with advanced malignancies [32].

### Imipridones target mitochondrial energy metabolism through activation of CLPP

The imipridone ONC201 (also known as TRAIL-inducing-compound 10, TIC10) was discovered in an attempt to identify drugs that are capable of inducing TRAIL, which was once considered as a potential “holy grail” of anti-cancer therapy [33]. While normal cells are not affected by TRAIL, a significant number of cancer cells display some or full susceptibility to this endogenous death ligand. The susceptibility of tumor cells towards TRAIL is determined by the expression levels of death receptors, c-FLIP, the inhibitors of apoptosis proteins, the Bcl-2 family members of proteins and the activity of NF-κB signaling. Thus, it is not surprising that numerous combination therapies, involving TRAIL that interfere with these pathways/targets were proposed over the last two decades. Early experiments from 1999 indicate that TRAIL was capable of prolonging overall survival in the U87 GBM model system *in vivo* [34]. While TRAIL had an impact on survival in this model system the benefit was not durable. To this end, a couple of years later TRAIL was combined with SMAC (second mitochondrial activator of caspases)-peptides, which are compounds that mimic the function of SMAC/DIABLO [35]. At the time it was assumed that this protein predominantly interferes with the so-called inhibitor of apoptosis proteins, such as XIAP and others, which interfere with the activity of caspases and thereby function as another fundamental regulator of apoptosis aside from the Bcl-2 family of proteins. In these experiments, the combination treatment led to long-haul survival of the animals [35]. Despite the promise there appear to be challenges in the translation of these findings to patients. As mentioned, imipridones were expected to address this issue. Recent studies suggested that imipridones induced cell death in a variety of cancer cells, including GBM, which involved *in vitro* but more relevantly *in vivo* experiments, including an orthotopic model of GBM. Mechanistically, the upregulation of TRAIL in cancer cells was mediated in part via the transcription factor, FOXO3, through a cascade of phosphorylation reactions, involving the kinases AKT and ERK. Akin to the death ligand itself imipridones synergized with a number of drugs as anticipated. For instance, the earlier concept that combined activation of both extrinsic and intrinsic apoptosis leads to substantial GBM cell killing was confirmed as well. In this regard, in utilizing the BH3-mimetic, ABT263, our group was able to demonstrate that loss of function of Bcl-xL combined with imipridones elicited synergistic reduction of cellular viability in a number of different GBM cells as well as in a proneural mouse model *in vivo* [36]. Initially, imipridones were thought to primarily interfere with AKT and ERK, but it was found later that they seem to primarily target a mitochondrial protease, called CLPP. Knockdown of CLPP in both acute myeloid leukemia and GBM cells rescued from the loss of viability induced by imipridones [37, 38]. Moreover, treatment resistance towards imipridones was in part mediated by the emergence of a CLPP mutation (D190A). The activation of CLPP by imipridones caused a depletion of enzymes involved in cellular respiration and potentially other mitochondrial proteins [39]. While many compounds exert effects on metabolism there is one important aspect with regards to imipridones, which relates to the fact that indeed imipridones kill cancer cells dependent on the loss of function of cellular energy metabolism, indicating that their impact on metabolism is not merely a passenger effect [40]. Consequently, low glucose conditions which favor oxidative energy metabolism rendered GBM cells more sensitive to the cytotoxic actions of ONC201 [40].

### A novel synergistic combination therapy, involving imipridones and HDAC inhibitors in GBM

Our group has recently found that FDA-approved HDAC-inhibitors may have a profound impact on energy metabolism in solid tumor cells, including GBM. While HDAC-inhibitors suppressed glycolysis in part through interference with enhancers they activated cellular respiration, which was fueled by fatty acid oxidation and was mediated by transcription factors related to either oxidative metabolism or fatty acid oxidation, e.g., PGC1A and PPARD [14]. This metabolic reprogramming appeared to become more prominent over time since GBM cells chronically exposed to HDAC blockers increased their oxygen consumption rate stronger than cells treated only short-term [14]. Because of the impact of HDAC-inhibitors on metabolism we hypothesized that imipridones, which suppress cellular respiration, might synergize with these compounds to significantly enhance killing of GBM cells [38]. Indeed, we found that imipridones reversed HDAC-inhibitor induced activation of cellular respiration and in turn the combination treatment facilitated induction of intrinsic apoptosis in a manner that was partially reliant on the anti-apoptotic Bcl-2 family members. In an orthotopic GBM xenograft model the combination treatment of imipridones and HDAC-inhibitors resulted in an increased survival as well, suggesting potential translational relevance [38]. In summary, targeting tumor cell metabolism is relevant and future research needs to identify patient populations that particularly benefit from such treatments. Moreover, while most studies related to metabolism still rest predominantly on the tumor cells it is critical to extend such observations to the microenvironment of the tumors, especially with regards to the immune system [41, 42].
